# Association between Interferon Response and Protective Efficacy of NS1-Truncated Mutants as Influenza Vaccine Candidates in Chickens

**DOI:** 10.1371/journal.pone.0156603

**Published:** 2016-06-03

**Authors:** Hyesun Jang, John M. Ngunjiri, Chang-Won Lee

**Affiliations:** 1 Food Animal Health Research Program, Ohio Agricultural Research and Development Center, The Ohio State University, Wooster, OH 44691, United States of America; 2 Department of Veterinary Preventive Medicine, College of Veterinary Medicine, The Ohio State University, Columbus, OH 43210, United States of America; Sun Yat-sen University, CHINA

## Abstract

Influenza virus mutants that encode C-terminally truncated NS1 proteins (*NS1-*truncated mutants) are attractive candidates for avian live attenuated influenza vaccine (LAIV) development because they are both attenuated and immunogenic in chickens. We previously showed that a high protective efficacy of *NS1-truncated* LAIV in chickens corresponds with induction of high levels of type I interferon (IFN) responses in chicken embryonic fibroblast cells. In this study, we investigated the relationship between induction of IFN and IFN-stimulated gene responses *in vivo* and the immunogenicity and protective efficacy of *NS1-truncated* LAIV. Our data demonstrates that accelerated antibody induction and protective efficacy of *NS1-truncated* LAIV correlates well with upregulation of IFN-stimulated genes. Further, through oral administration of recombinant chicken IFN alpha in drinking water, we provide direct evidence that type I IFN can promote rapid induction of adaptive immune responses and protective efficacy of influenza vaccine in chickens.

## Introduction

Avian influenza viruses (AIVs) pose a constant threat to the poultry industry with avian influenza (AI) outbreaks resulting in significant economic losses [1–5]. During the 2015 highly pathogenic avian influenza (HPAI) outbreak in the Midwest, more than 40 million birds were killed and 10% of the US egg supply was affected [6]. In addition to their devastating impact on the poultry industry, occasional direct transmission of AIVs from poultry to humans has resulted in serious outbreaks in the past that produced fatal outcomes [7, 8]. The recent avian H5N1, H7N7, and H7N9 human outbreaks in China and Europe have come with severe respiratory illness resulting in severe respiratory symptoms and death in some cases [8–10].

Avian influenza can be prevented, managed, or eradicated through programs that focus on education, diagnostics, surveillance, biosecurity, elimination of infected poultry, and reduction of host susceptibility to AIVs [11]. While pre-emptive culling of affected flocks is the most preferred method of controlling the spread of HPAI virus during an outbreak, it inevitably results in huge monetary losses. Such losses can be prevented by decreasing host susceptibility through vaccination or, in the event of an outbreak, by selective culling followed by vaccination.

Whole inactivated virus influenza vaccines are the most commonly used vaccines in poultry [12]. Although these vaccines provide excellent protection from homologous strains, they are less effective or completely unprotective against heterologous and heterosubtypic strains. In addition, the inactivated vaccines do not elicit strong cross-reactive T-cell and mucosal immune responses. Clearly, broadly protective AI vaccines need to be developed.

Novel influenza vaccine designs seek to increase the breadth of heterologous and heterosubtypic cross-protection. One approach is to develop inactivated vaccines that selectively induce broadly neutralizing antibodies that target the conserved regions of viral proteins, such as HA stalk or the ectodomain of M2 protein (M2e) [13, 14]. Another approach is to use live attenuated influenza vaccines (LAIV) with capacities to elicit long lasting immunity by stimulating mucosal, cellular, and systemic (IgG) responses that are cross protective against heterologous and heterosubtypic viral infections [11–14].

The nonstructural protein 1 [NS1] of influenza virus has been an attractive target for attenuation in LAIV development strategies. The NS1 protein is known to enhance virus replication by antagonizing antiviral host cell functions, especially by blocking type I interferon (IFN) responses [15]. In this context, influenza viruses with truncation in the NS1 (*NS1-truncated*) provoke high type I IFN responses and replicate poorly in IFN competent hosts [16]. However, we have observed that not all *NS1-truncated* variants are effective as LAIV candidates [17]. Four *NS1-truncated* mutants were previously tested for their capacity to induce protective immunity in chickens [17]. Two of the mutants (pc3-LAIV and pc4-LAIV) were more efficacious than the other two (pc1-LAIV and pc2-LAIV) in protecting chickens against heterologous challenge virus [17].

A series of *in vitro* experiments were subsequently carried out to establish why these LAIV candidates differ in their protective efficacy [18, 19]. The *in vivo* efficacy of vaccine candidates [17] was observed to correlate strongly with induction of high yields of type I IFN *in vitro* [18, 19]. For example, infection of chicken embryonic fibroblasts with pc4-LAIV, the more efficacious LAIV in chickens, resulted in production of high levels of type I IFN compared to pc2-LAIV (the less effective vaccine) [17, 18]. This finding is suggestive but does not prove that type I IFN is required to boost the efficacy of *NS1-truncated* LAIV in chickens.

In the current study, we sought to establish the relationship between the induction of IFN and IFN-stimulated gene (ISG) responses *in vivo* and the immunogenicity and protective efficacy of *NS1-truncated* LAIV. Our data demonstrates that the level of antibody induction and protective efficacy of *NS1-truncated* LAIV correlates well with upregulation of ISG expression. Further, through oral administration of recombinant chicken IFN alpha (rChIFN-α) in drinking water, we provide direct evidence that type I IFN is a potent adjuvant for influenza vaccine in chickens.

## Materials and Methods

### Animals and ethics statement

All animals were maintained, vaccinated, challenged and euthanized in accordance with protocol #2009AG0002-R2 approved by The Ohio State University Institutional Animal Care and Use Committee (IACUC). This protocol complies with the U.S Animal Welfare Act, Guide for Care and Use of Laboratory Animals and Public Health Service Policy on Humane Care and Use of Laboratory Animals. The Ohio State University is accredited by the Association for the Assessment and Accreditation of Laboratory Animal Care International (AAALAC). White leghorn chickens were obtained from our institutional (Food Animal Health Research Program, Wooster, OH) specific pathogen free (SPF) flock. The chickens were housed in a BSL2 facility with forced air ventilation and adequate air exchanges to prevent ammonia build up. Air entering or leaving the facility is HEPA filtered. The birds were kept in large cages (2592 sq. inch) before infection and transferred to Model 934–1 isolators (900 sq. inch) (Federal Designs Inc., Comer, GA). The number of birds in each cage was calculated based on age and the space available after subtracting the space occupied by the feeder and the watering system. Room and isolator temperatures were maintained at 25±3°C. Birds had *ad libitum* access to feed and water. The wellbeing and health status of the animals was monitored twice daily throughout the experiments. Animals were humanely euthanized when they displayed symptoms such as ruffled feathers and reluctance to move, not moving when prodded, respiratory distress, or injuries that were not related to experimental treatment. Euthanasia was actualized by exposure to carbon dioxide (CO_2_). Based on the age and body size, 1–10 animals were placed in the euthanasia chamber connected to a CO_2_ source. The CO_2_ flow was set at 10–30% displacement of chamber volume/minute. Birds were observed for respiratory arrest and the CO_2_ flow was maintained for at least one minute after the arrest was observed. The animals were checked for an absence of breathing and lack of heartbeat. If any respiration or heartbeat was detected, the animal was placed back into the chamber and additional CO_2_ was administered as described above. After death has been confirmed, an additional secondary physical euthanasia (cervical dislocation or removal of a vital organ) was performed before collection of tissues and carcass disposal.

### Vaccination with live-attenuated influenza vaccine (LAIV) candidates

Groups of four-week-old SPF chickens (n = 23 per group) were intranasally mock-vaccinated with phosphate-buffered saline (PBS) or intranasally inoculated with *NS1-truncated* LAIV candidates (pc2-LAIV and pc4-LAIV, both derived from wildtype virus A/TK/OR/71 (H7N3)) or reverse genetically created wildtype A/TK/OR/71 (H7N3) (rgWT) virus [17, 20] at a dose of 10^6^ EID_50_ per bird. Five birds per group were euthanized at 1, 2, and 3 days post-inoculation (dpi) to harvest trachea and spleen tissues for analysis of gene expression. The remaining 8 birds per group were bled at 8 and 14 dpi for detection and titration of hemagglutination-inhibition (HI) antibodies [21].

### Oral recombinant chicken IFN-α (rChIFN-α) treatment and vaccination with inactivated influenza vaccine

Cloning and expression of rChIFN-α in mammalian cells was described previously [22]. Groups of four-week-old SPF chickens (n = 20 per group) were mock-vaccinated or subcutaneously vaccinated with PBS or whole-inactivated rgWT virus vaccine and provided with plain drinking water or drinking water with rChIFN-α (10^5^ Units/bird/day). The inactivated vaccine was prepared by treating the rgWT virus with betapropiolactone as previously described [23]. Five birds per group were euthanized at 1, 3, and 8 days post start of rChIFN-α treatment (dpt) for transcription analysis. All of the remaining birds (10 birds/group at 8 dpt and 5 birds/group at 14 dpt) were bled for detection and titration of HI antibodies.

### Oral rChIFN-α or Poly I:C treatment and vaccination with NS1 variants

Four-week-old SPF chickens (n = 35) were divided into 5 groups: 1. unvaccinated control; 2. pc2-LAIV vaccinated; 3. pc2-LAIV vaccinated + per-oral rChIFN treated; 4. pc2-LAIV vaccinated + per-oral treated with high molecular weight (1.5–8 kb) VacciGrade polyinosinic-polycytidylic acid (poly(I:C)) (InvivoGen); and 5. pc4-LAIV vaccinated. Vaccination with live virus and oral treatment with rChIFN-α were conducted as described above. At 14 dpv, all chickens were challenged with a heterologous strain A/CK/NJ/150383-7/02 (H7N2), and the replication of challenge virus was evaluated from tracheal swab samples collected at 2 and 4 days post challenge (dpc).

### Transcriptional analysis

Total RNA was extracted from trachea and spleen tissues using Trizol and subjected to quantitative reverse transcription PCR (qRT-PCR) as previously described [24]. The primer sets used in this study were published previously [25]. The fold-change in gene expression was calculated using the ΔΔCt method using GAPDH gene as the internal control [26]. All groups were included in the statistical analyses where the unvaccinated (uninfected) and untreated control groups were used as references. To plot the figures, the expression fold change value was normalized by dividing with that of the corresponding gene in the control group. Therefore, the normalized fold change of each gene in the control group is 1.

### Virus replication in chickens

Tracheal swabs were collected at the indicated time points and eluted in 1 ml of PBS supplemented with gentamicin (10 μg/ml) for virus detection. RNA was extracted from 100 μl of the sample using QIAamp Viral RNA Mini Kit (Qiagen). The remaining sample was used for virus isolation. To allow interpolation of median egg infective dose (EID_50_) titers of swab samples by the qRT-PCR method [27, 28], a standard curve was created by plotting cycle threshold (Ct) values generated with RNA extracted from serial 10-fold dilutions of the same virus stock (with known EID_50_ titer) used to inoculate the chickens as a function of virus dilution. The curve was used to convert Ct values of tracheal swab viral RNA to EID_50_ titers. EID_50_ titers derived by qRT-PCR are equivalent to EID_50_ titers measured in eggs [28]. MDCK cells were used for virus isolation and median tissue culture infective dose (TCID_50_) calculation. The cells were propagated in DMEM (Dulbecco's Modified Eagle Medium) supplemented with 10% fetal bovine serum (FBS) and 10 μg/ml gentamicin. Serial 10-fold dilutions of tracheal swab eluate were prepared in serum-free DMEM containing 0.75 μg/ml TPCK trypsin. Confluent cell monolayers in 96-well tissue culture plates were washed two times with PBS, inoculated with 100 μl of the diluted sample (5 replicate wells per dilution), and incubated for 5 days at 37°C. Hemagglutination assay was used to detect virus in the supernatant medium. TCID_50_ was then calculated by the Reed-Muench method [29].

### Statistical analysis

The Mann-Whitney U test (SPSS software) was used to determine differences between transcriptional fold-change values. Differences in virus titers among groups were determined by the One-way ANOVA, followed by post-hoc Tukey test (GraphPad Prism version 5.00 for Windows (GraphPad Software, San Diego California USA)) for the pair-wise comparison.

## Results

### Serum antibody response in chickens vaccinated with experimental *NS1-truncated* LAIV

**Fig 1:**
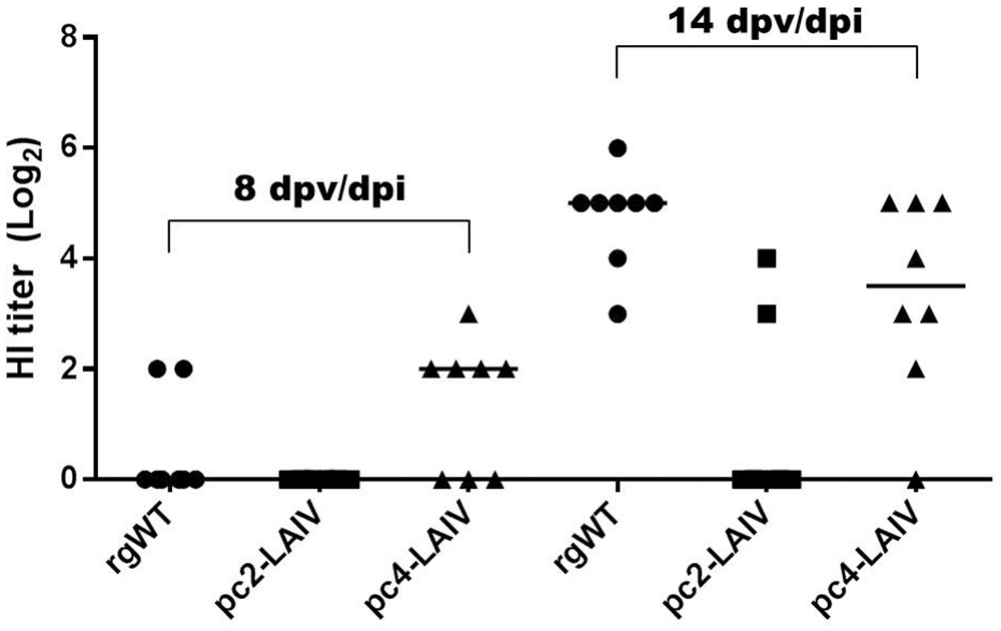
Serum antibody response in chickens following vaccination with LAIV candidates or infection with rgWT virus. Serum was collected at 8 and 14 days post-vaccination/infection (dpv/dpi) and tested for the presence of influenza virus A/TK/OR/71 specific hemagglutination inhibition (HI) antibodies. HI titers are presented as circles (rgWT), squares (pc2-LAIV), and triangles (pc4-LAIV). The thick horizontal lines represent median titers of the groups.

We first looked at the development of adaptive immune responses in 4-week-old chickens (n = 8 per group) after intranasal vaccination with pc2-LAIV and pc4-LAIV. The reverse genetically created wildtype (rgWT) virus was included for comparison. Among birds vaccinated with pc4-LAIV, five had detectable levels of HI antibodies as early as 8 days post-vaccination/infection (dpv/dpi) (Fig 1). This was in clear contrast with pc2-LAIV vaccination where none of the birds were HI positive or rgWT infection where only 2 birds had detectable antibodies at this time point. At 14 dpv/dpi, only two birds in the pc2-LAIV-vaccination group were HI positive compared to 7 in the pc4-LAIV-vaccination group and 8 in the rgWT-infected group.

**Fig 2:**
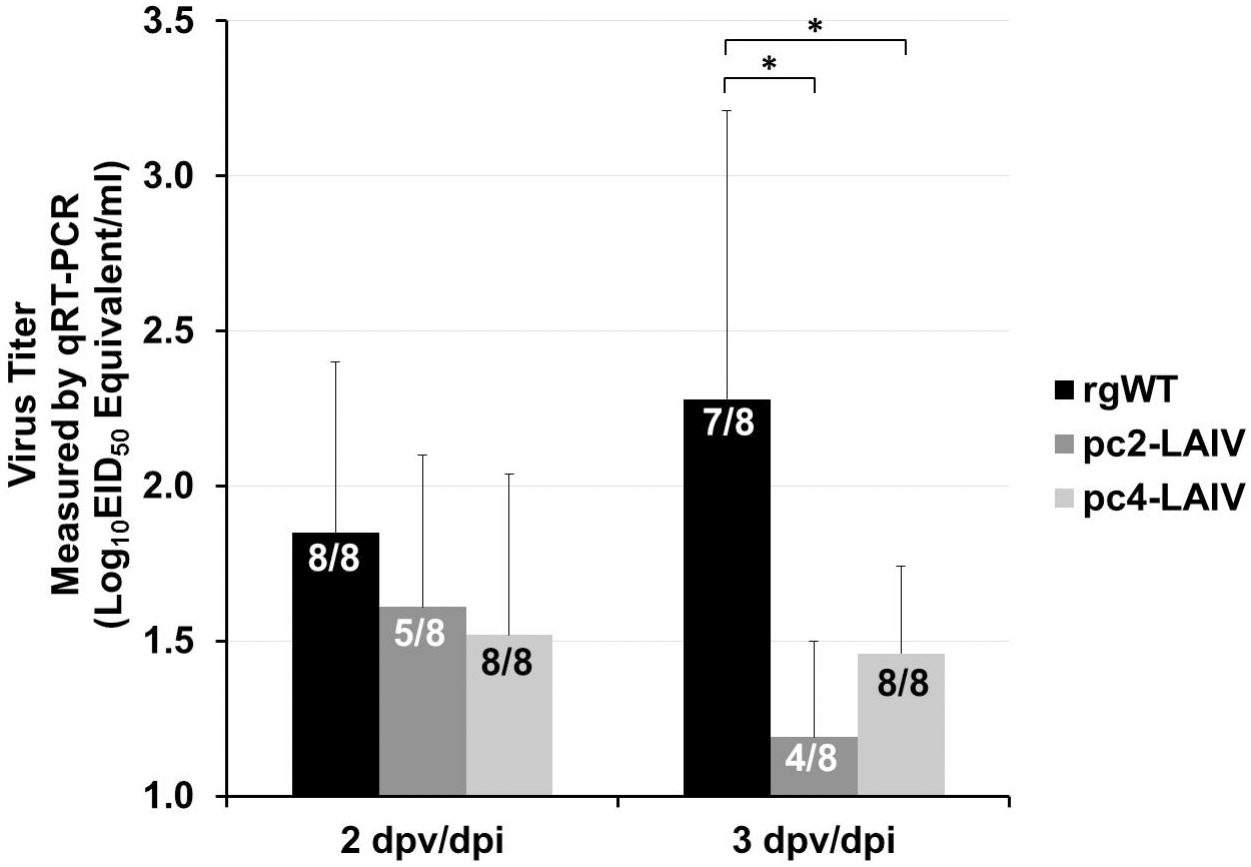
Comparison of virus replication in trachea. The EID_50_ equivalent titers were interpolated from qRT-PCR Ct values of tracheal swab viral RNA as described in Materials and Methods. Statistical significance, *p<0.05. EID_50_, median egg infectious doses. *n/n*, number of virus positive birds/total number of birds in the group.

To rule out the possibility that the rapid and high ratio of seroconversion in the pc4-LAIV-vaccination group was due to high replication efficiency (or high antigen load) of the vaccine virus, viral titers were determined from tracheal swabs. Birds in all three groups had similar viral titers at 2 dpv/dpi (Fig 2). At 3 dpv/dpi, the rgWT viral titers were significantly higher than pc2-LAIV or pc4-LAIV by several folds and there was no difference between pc2-LAIV and pc4-LAIV even though not all the birds in the pc2-LAIV group were PCR positive (Fig 2).

### Gene expression in chickens vaccinated with experimental *NS1-truncated* LAIV

**Fig 3:**
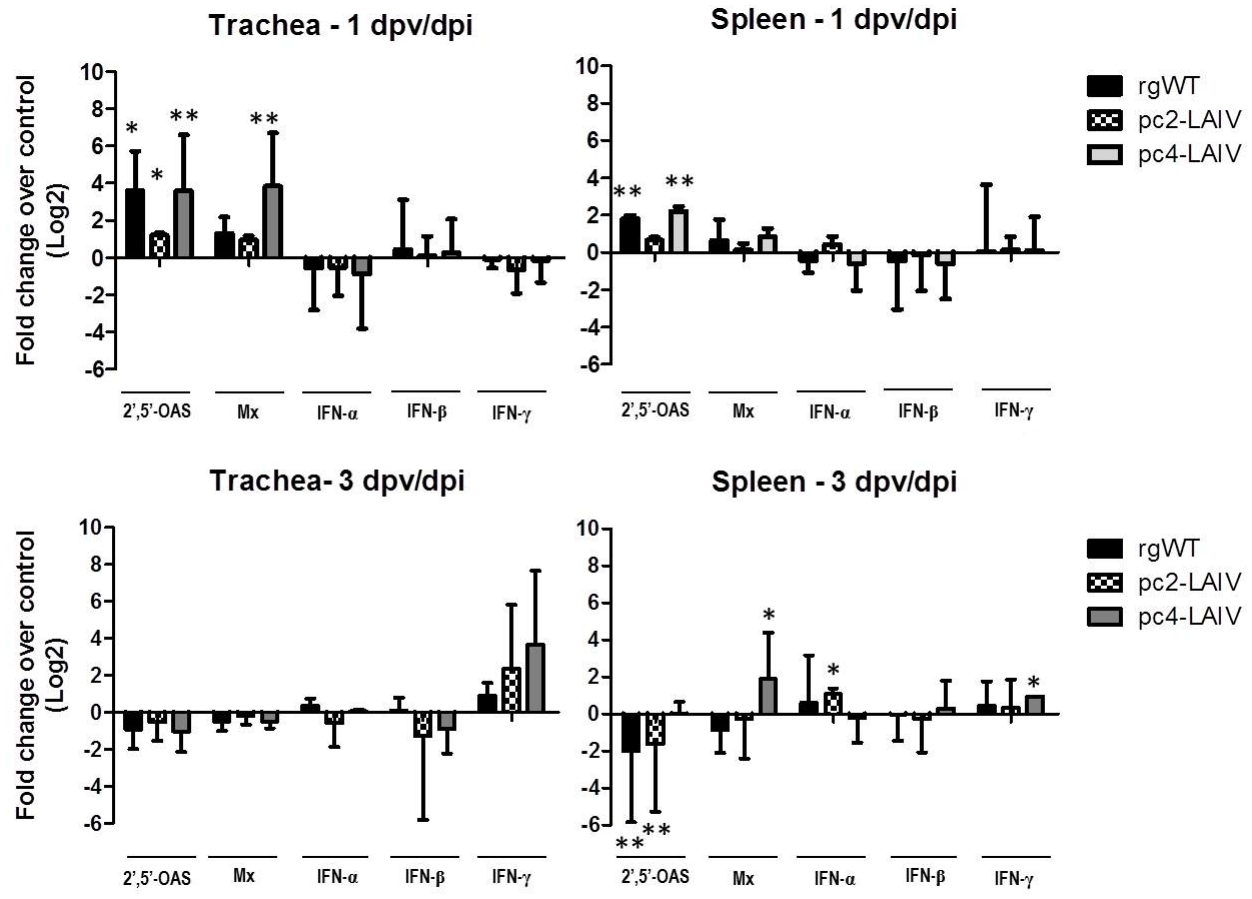
IFN and ISG responses after vaccination with LAIV candidates or infection with rgWT virus. See Materials and Methods for details on fold change calculation, statistical analysis and data normalization. dpv/dpi, days post vaccination/infection. All groups were included in the statistical analysis where the unvaccinated (uninfected) group was used as the reference. Error bars, mean ± S.D. Statistical significance, *p<0.05, **p<0.001.

In a previous *in vitro* study, pc4-LAIV induced a higher level of type I IFN response in chicken embryonic fibroblasts than pc2-LAIV and rgWT virus [18]. Thus, the rapid seroconversion of pc4-LAIV-vaccinated chickens (Fig 1) could be due to high IFN response *in vivo*. We tested this possibility by analyzing the expression levels of IFN and IFN-related genes in trachea and spleen tissues following vaccination with LAIV candidates or infection with rgWT virus. Two IFN-stimulated genes (ISGs), 2′,5′-OAS and Mx, were targeted due to their high sensitivity to the type I IFN stimulus. Fig 3 shows that at 1 dpv/dpi, the level of 2′,5′-OAS gene transcription was significantly increased in trachea and spleen tissues of chickens vaccinated with pc4-LAIV or infected with rgWT but not in the trachea of pc2-LAIV-vaccinated birds. Expression of the Mx gene was significantly upregulated only in 1 dpv tracheal tissues from pc4-LAIV-vaccinated chickens and downregulated in 3 dpv/dpi spleen samples from pc2-LAIV and rgWT vaccinated/infected birds. We could not detect any increase in type I IFN (IFN-α/β) gene transcription although there was upregulation of the IFN-γ gene in spleen samples collected from the pc4-LAIV-vaccincation group at 3 dpv.

### Oral IFN treatment induces a rapid antibody response to influenza vaccination in chickens

**Fig 4:**
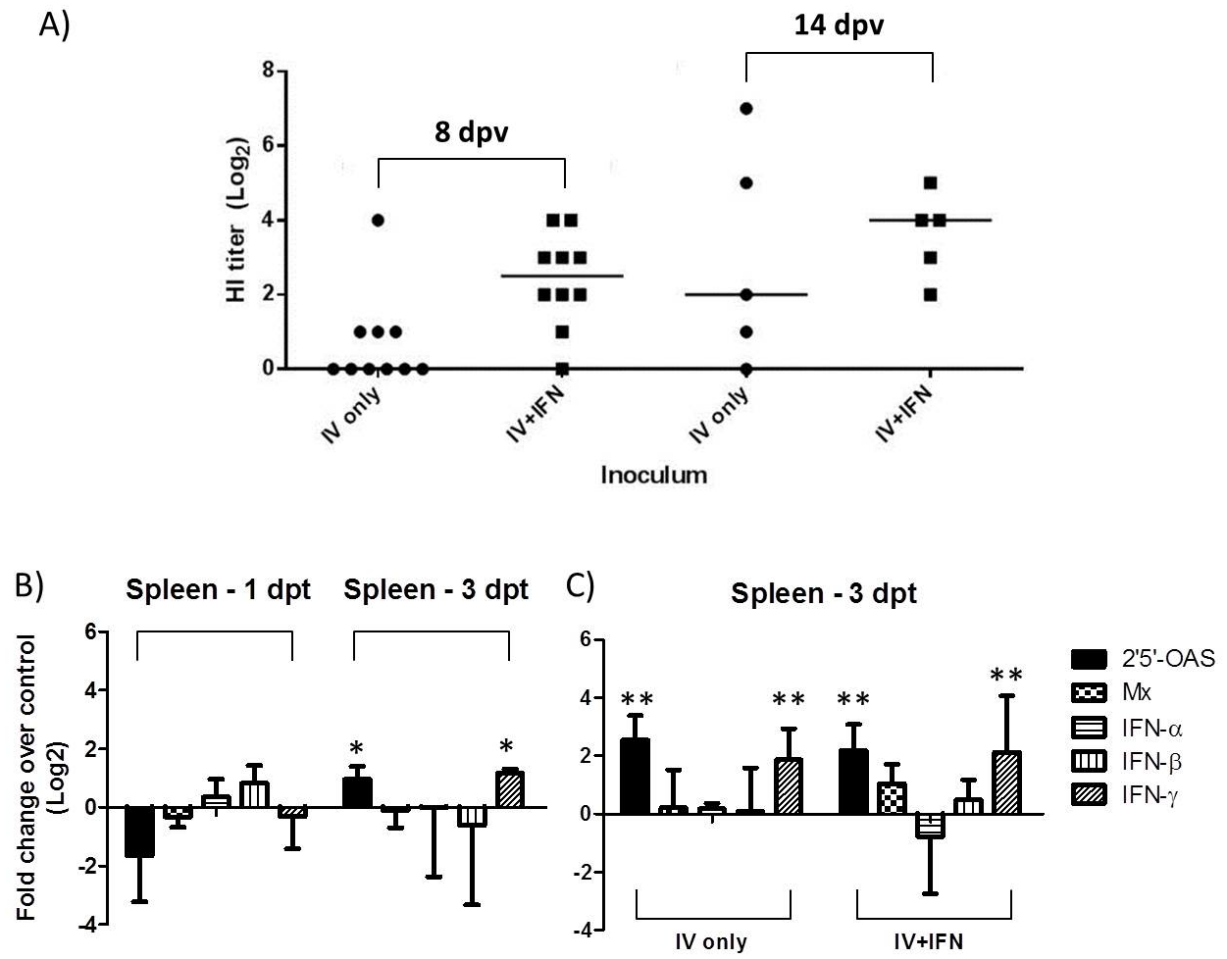
Oral rChIFN-α treatment in chickens. (A) Antibody response to vaccination with inactivated vaccine (IV) with or without rChIFN-α (IFN) treatment at 8 and 14 days post vaccination (dpv). (B) ISG and IFN gene responses in spleens of unvaccinated chickens at 1 and 3 days post treatment (dpt). (C) Comparison of ISG and IFN gene responses in spleens of chickens vaccinated with IV with or without rChIFN-α treatment at 3dpt. All groups were included in the statistical analysis where the untreated control group was used as the reference. Statistical significance, *p<0.05, **p<0.001.

The observed correlation between antibody response and transcription of IFN-related genes in pc4-LAIV-vaccinated birds (Figs 1 and 3) is suggestive but does not prove that IFN is involved in rapid seroconversion. To assess the direct role of IFN in stimulating rapid seroconversion further, we subcutaneously inoculated chickens with inactivated influenza vaccine and provided them with rChIFN-α in drinking water at an average dose of 10^5^ Units/bird/day for 14 days. This dose of type I IFN was previously shown to have biological effects in 33-day old chickens [30]. Fig 4A shows that 9 of 10 rChIFN-α-treated birds seroconverted by 8 days dpt compared to only 4 out of 10 birds provided with plain drinking water. All remaining birds (n = 5) in the rChIFN-α-treatment group seroconverted by 14 dpt while one of the 5 birds provided with plain drinking water was HI negative. Further, a wider range of HI titers was observed in the untreated group (Log_2_ HI titer <1–7) relative to the treated birds (Log_2_ HI titer 2–6) at 14 dpt (Fig 4A). The rapid seroconversion of rChIFN-α-treated chickens is similar to that observed in the pc4-LAIV vaccination group (Fig 1).

### Gene expression in chickens treated with rChIFN-α in drinking water

Biological activity of orally-administered rChIFN-α was previously reported to correlate with increase in the level of Mx1 and 2′,5′-OAS gene expression in trachea tissues [30]. In this study, we focused on the effect of oral rChIFN-α treatment on the expression of these two ISGs along with type I IFN (IFN-α/β) and IFN-γ genes. As shown in Fig 4B, treatment with rChIFN-α resulted in significant upregulation of 2′,5′-OAS and IFN-γ genes in spleen tissues harvested from unvaccinated chickens at 3 dpt. The rChIFN-α treatment did not cause upregulation of 2′,5′-OAS, Mx1, and IFN-γ gene expression in spleen at 1 dpt. While the transcription levels of 2′,5′-OAS and IFN-γ genes were upregulated in birds administered with inactivated vaccine, there was no statistical difference between rChIFN-α-treated and untreated birds (Fig 4C).

### Effect of type I IFN treatment on immunogenicity and heterologous protection efficacy of pc2-LAIV

**Fig 5:**
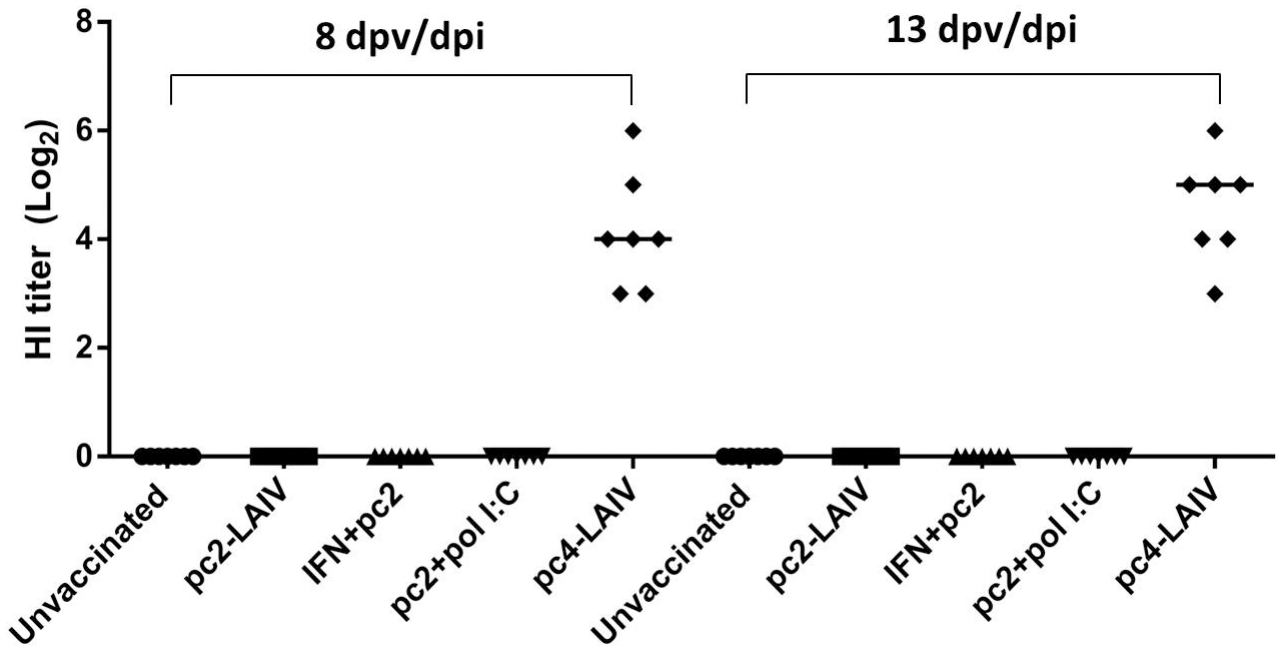
Pre-challenge antibody responses. The development of antibody response was monitored at 8 and 13 days post-vaccination (dpv). Horizontal bars represent mean antibody titer for the group (*n = 7*).

**Fig 6:**
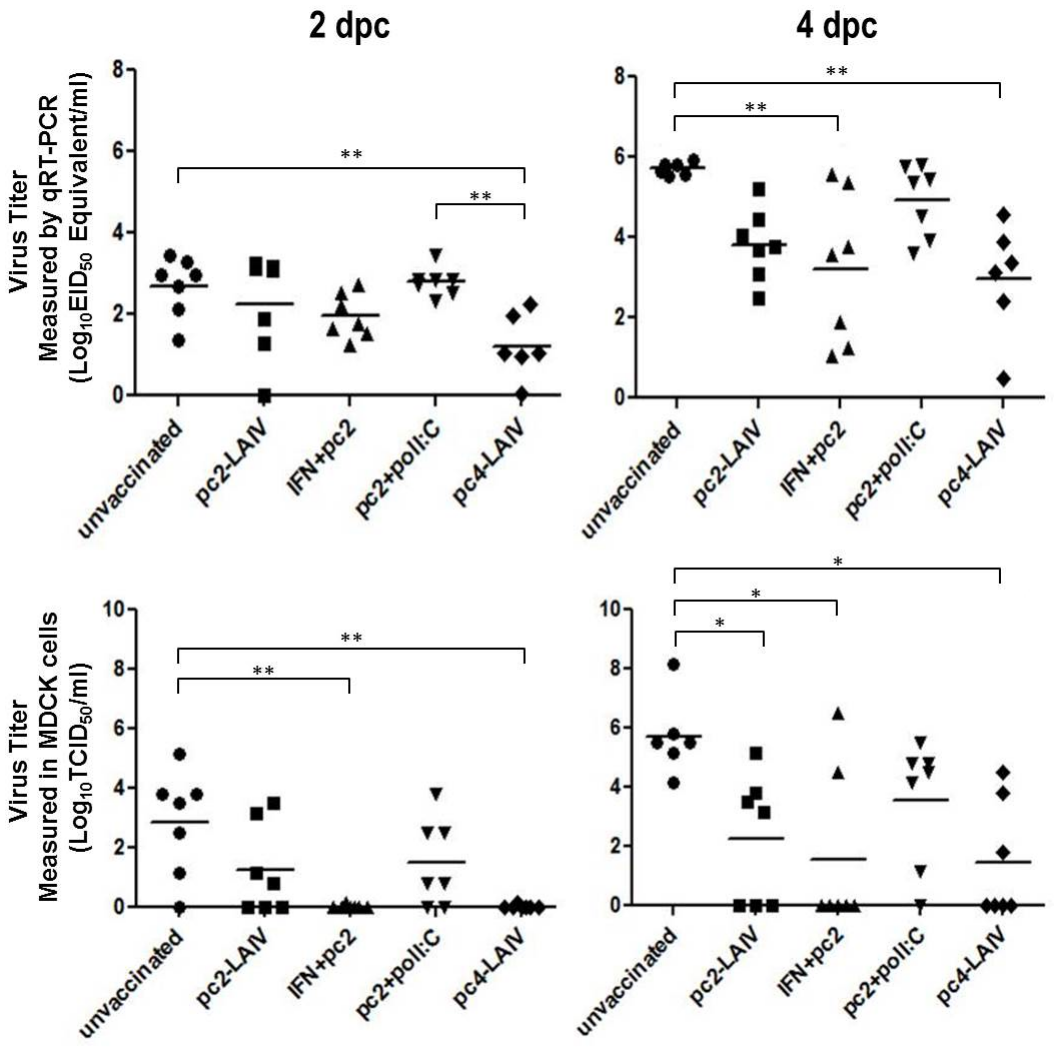
Replication and shedding of heterologous challenge virus. Top: Viral titers expressed as median (50%) egg-infectious dose equivalent by qRT-PCR (see Materials and Methods) [27, 28]. Bottom: viral titers re-isolated in MDCK cells. Horizontal bars represent mean antibody titer for the group (*n = 7*). dpc, days post challenge. TCID_50_, median (50%) tissue culture infective dose. *p<0.05. **p<0.001.

Aforementioned data suggests that immunogenicity of *NS1-truncated* LAIV candidates is partially dependent on the levels of type I IFN induced *in vivo*. Thus, we hypothesized that the poor immunogenicity and inefficacy of pc2-LAIV is mainly due to the lower type I IFN induction capacity in chickens and tested whether oral treatment with type I IFN can boost the protective efficacy of pc2-LAIV. Thirty five chickens were divided into five groups (n = 7 per group): unvaccinated control; pc2-LAIV vaccination; IFN treatment + pc2-LAIV vaccination (exogenous rChIFN-α treatment); poly I:C treatment + pc2-LAIV LAIV vaccination (endogenous IFN induction); and pc4-LAIV vaccination groups. Poly I:C was previously shown to enhance adaptive immune responses to influenza vaccine in chickens [31]. Vaccine immunogenicity was first assessed by testing serum HI antibody titer. As described above (Fig 1), pc4-LAIV vaccination provoked an early antibody response at 8 dpv (5 out of 8 birds (65.5%)), but no antibody response was detected in pc2-LAIV-vaccinated animals even after treatment with exogenous rChIFN-α or poly I:C (Fig 5). At 15 dpv, we challenged the birds with a heterologous (H7N2) virus and compared the protective efficacy among the vaccination groups. One bird in the unvaccinated control group was euthanized at 2 days post challenge (dpc) due to severe clinical symptoms (ruffled feathers and periorbital swelling). Consistent with the previous report [17], pc4-LAIV vaccination consistently showed the highest degree of protection against the heterologous challenge virus as indicated by EID_50_ equivalent titers detected by qRT-PCR (Fig 6, top (*p<0*.*001*)) and confirmed by virus isolation in MDCK cells at 2 and 4 dpc (Fig 6, bottom (2 dpc, p*<0*.*001*; 4 dpc, *p<0*.*05*)). In contrast, significant reduction of virus shedding by pc2-LAIV vaccination was only detected in 4 dpc samples using the virus isolation method (Fig 6, bottom right (*p<0*.*05*)). Of note, treatment of birds with rChIFN-α prior to pc2-LAIV vaccination led to a significant reduction in the titer of re-isolated challenge virus at both time points (Fig 6, bottom (2 dpc, *p<0*.*001*; 4 dpc, *p<0*.*05*)) and virus detected by qPCR at 4 dpc (Fig 6, top right (*p<0*.*001*)). Poly I:C treatment did not enhance the efficacy of pc2-LAIV, rather it appears to have increased the level of virus replication (Fig 6).

## Discussion

We have compared two *NS1-truncated* mutants in terms of their immunogenicity, ability to induce IFN and ISG responses, and protective efficacy in four-week-old chickens. During the first two weeks post vaccination, the development of adaptive immune responses was monitored by measuring HI antibody titers in serum samples. In line with our previous observation [17], pc4-LAIV was superior to pc2-LAIV in terms of inducing seroconversion and HI antibodies in chickens. In addition, the current study has provided new insight into induction of adaptive immune responses by *NS1-truncated* LAIV candidates. We have demonstrated that pc4-LAIV consistently induced a rapid antibody response within 8 days following intranasal vaccination (Figs 1 and 5). This can shorten the risk period between vaccination and the development of protective immunity especially in young birds that do not respond well to inactivated vaccines [32]. The inability of pc2-LAIV to induce seroconversion may be attributed to over-attenuation (suboptimal replication) (Fig 1) and poor immunogenicity of the vaccine.

In general, *NS1-truncated* mutants are attenuated in avian and mammalian species partly due to induction of high type I IFN responses [16, 33]. The type I IFN is also known to enhance the mucosal and systemic adaptive immune responses [34–36]. In chickens, rChIFN-α treatment was shown to induce more rapid seroconversion to natural infection by low-pathogenicity influenza virus [37]. In mice, IFN-α/β treatment promoted fast and polyclonal antibody responses [38] and a recombinant rabies virus expressing IFN-α1 was shown to stimulate an antibody response that was more rapid compared to the isogenic wildtype virus [39]. Thus, induction of rapid immune responses by the pc4-LAIV may be due to its capacity to trigger higher levels of type I IFN compared to pc2-LAIV and rgWT virus [18].

Contrary to the high levels of type I IFN induced in primary chicken fibroblast cells [18], expression of IFN-α/β genes was generally not upregulated except for a small but statistically significant increase in IFN-α transcription in 3 dpv spleens of the pc2-LAIV group (Fig 3). The discrepancy between *in vitro* and *in vivo* IFN inducing capacities of our vaccine candidates is a subject for future study. Penski et al [40] reported a similar discrepancy where a set of *NS1-truncated* mutants were able to induce high levels of IFN in chicken cell cultures but were poor inducers in chickens, *in vivo*, in a manner that correlated with virus replication. The fact that our vaccine candidates and the isogenic rgWT virus are very attenuated in chickens (<10^3^ EID_50_/ml of swab eluate) (Fig 1) could explain why we did not see upregulation of IFN genes in trachea and spleen tissues collected at 1 and 3 dpv/dpi. However, it does not explain why there was significant upregulation of the 2′,5′ OAS gene in trachea and spleen tissues of chickens vaccinated with pc4-LAIV or infected with rgWT and Mx gene in tracheal tissues from pc4-LAIV-vaccinated chickens (Fig 3). It is possible that both pc4-LAIV and rgWT were able to induce some IFN that triggered ISG upregulation [41]. We could have missed a critical time point for IFN-α/β gene detection or a cell population that produces large amounts of the cytokine in chickens [40]. Alternatively, ISG transcription may have been triggered directly by the virus infection independently of IFN signaling [42, 43]. For example, the 2′,5′-OAS gene can be activated by dsRNA independently of IFN signaling [43]. The level of ISG transcription can also be affected by the ability of truncated or full-size NS1 proteins to suppress epigenetic control of gene regulation [44, 45]. Although our study focused on the 2′,5′-OAS and Mx genes, there are more than 300 ISGs [46]. An in-depth study is required to identify ISGs that are critical for vaccine efficacy and to delineate the mechanism of ISG upregulation by the *NS1-truncated* LAIV candidates.

We reasoned that if the rapid seroconversion triggered by pc4-LAIV was due to ISG upregulation, a similar response could be produced through rChIFN-α treatment. As observed in the study published by Meng *et al*. [30], rChIFN-α treatment resulted in elevated levels of 2′,5′-OAS gene expression in spleen at 3 dpt (Fig 4B). Thus, our orally-administered rChIFN-α was biologically active. Clearly, the rapid antibody development in birds treated with rChIFN-α and vaccinated with whole inactivated rgWT virus vaccine (Fig 4A) was similar to that observed in pc4-vaccinated birds (Figs 1 and 5). It is worth noting that both the rgWT virus and pc4-LAIV have the same backbone genes and proteins except for the NS1 gene/protein [17] and humoral immune response to whole virus inactivated vaccine is mainly directed to HA and NA proteins (not the NS1 protein) [47]. Therefore, a whole inactivated pc4 vaccine is also expected to induce rapid seroconversion in rChIFN-α treated chickens. Future work should determine how ISGs, IFN, and pc4-LAIV interdependently or independently trigger the acceleration of adaptive immune responses.

The poor efficacy of pc2-LAIV may result from an inability to induce high levels of type I IFN [19] or ISGs (Fig 3). This prompted us to test whether direct (exogenous) IFN treatment or endogenous IFN induction by poly I:C can enhance pc2-LAIV efficacy. Heterologous protection by pc2-LAIV was significantly enhanced by rChIFN-α treatment (Fig 6). Although the *in vivo* half-life of rChIFN-α has yet to be determined, non-PEGylated interferons have short *in vivo* half-lives due to low stability. For example, in humans, orally administered non-PEGylated IFN has a half-life of up to 8.5 hours [48]. Since the rChIFN-α was administered for 4 days and withdrawn on the day of vaccination and the chickens were challenged at 2 weeks post vaccination, it is less likely that reduction of virus shedding was due to the innate effects of IFN.

The failure of pc2-LAIV to induce an antibody response when administered together with rChIFN-α is intriguing since rChIFN-α was previously shown to facilitate seroconversion of chickens after natural infection by low pathogenicity avian influenza virus [37]. The ability of IFN to facilitate seroconversion in the context of live virus may depend on the virus strain. The enhanced protective efficacy of pc2-LAIV in rChIFN-α treated chickens may be due to stimulation of cross-protective cell-mediated immunity [49–51]. We will address this possibility in a separate study. Another unexpected result was the observation that poly I:C treatment not only failed to enhance pc2-LAIV efficacy but also appeared to cause a slight increase in virus shedding at 4 dpc (Fig 6). We speculate that the dose of poly I:C (100 μg/bird) used in this study was not optimal for pc2-LAIV even though it was previously shown to enhance adaptive immune responses to inactivated avian H5N1 influenza vaccine in chickens [31].

In this study, we have demonstrated that the level of antibody induction and protective efficacy of *NS1-truncated* LAIV in chickens correlates well with upregulation of ISG expression. An in-depth analysis such as systems biology [52] is required to determine which ISGs need to be upregulated to enhance *NS1-truncated* LAIV efficacy.
